# Spatial Distribution of Non-Immune Cells Expressing Glycoprotein A Repetitions Predominant in Human and Murine Metastatic Lymph Nodes

**DOI:** 10.3390/cancers15235621

**Published:** 2023-11-28

**Authors:** Loïc Rouaud, Louis Baudin, Marine Gautier-Isola, Pierre Van Meerbeeck, Emilie Feyereisen, Silvia Blacher, Nicolas van Baren, Frédéric Kridelka, Sophie Lucas, Agnes Noel

**Affiliations:** 1 Laboratory of Tumor and Development Biology, GIGA, University of Liège (ULiège), Sart-Tilman, 4000 Liège, Belgium; loic.rouaud@uliege.be (L.R.); louis.baudin@uliege.be (L.B.); marinegautier06@gmail.com (M.G.-I.);; 2 Université Côte d’Azur, CNRS UMR7275, IPMC, 06560 Valbonne, France; 3 de Duve Institute, UCLouvain, 1200 Brussels, Belgiumnicolas.vanbaren@uclouvain.be (N.v.B.); sophie.lucas@uclouvain.be (S.L.); 4 Department of Obstetrics and Gynecology, CHU Liège, Sart-Tilman, 4000 Liège, Belgium; 5 WELBIO Department, WEL Research Institute, Avenue Pasteur, 6, 1300 Wavre, Belgium

**Keywords:** LRRC32, GARP mRNA, glycoprotein A repetitions predominant (GARP), transforming growth factor beta 1 (TGF-β1), lymph node, tumor microenvironment, metastases, cancer

## Abstract

**Simple Summary:**

Glycoprotein A repetitions predominant (GARP) is expressed at the surface of regulatory T lymphocytes (Tregs) in human and murine primary tumors and was shown to mediate TGF-β1 activation and immunosuppression by Tregs in tumor-bearing mice. The cellular sources and the implication of GARP in lymph nodes (LNs) during the metastatic cascade are still elusive. Here, we mined available scRNA-Seq datasets and conducted immunohistochemistry and in situ hybridization analyses of metastatic LNs from mice and patients with cervical or breast cancer. We found GARP expression not only in Tregs, but also in blood/lymphatic vessels, fibroblastic cells, and perivascular cells. Our study highlights for the first time GARP expression by specialized lymphatic endothelial cells in the subcapsular sinus, high endothelial venules (HEVs), and matrix-associated (fibroblastic/perivascular) cells.

**Abstract:**

Several types of cancer spread through the lymphatic system via the sentinel lymph nodes (LNs). Such LN-draining primary tumors, modified by tumor factors, lead to the formation of a metastatic niche associated with an increased number of Foxp3+ regulatory T cells (Tregs). These cells are expected to contribute to the elaboration of an immune-suppressive environment. Activated Tregs express glycoprotein A repetitions predominant (GARP), which binds and presents latent transforming growth factor beta 1 (TGF-β1) at their surface. GARP is also expressed by other non-immune cell types poorly described in LNs. Here, we mapped GARP expression in non-immune cells in human and mouse metastatic LNs. The mining of available (human and murine) scRNA-Seq datasets revealed GARP expression by blood (BEC)/lymphatic (LEC) endothelial, fibroblastic, and perivascular cells. Consistently, through immunostaining and in situ RNA hybridization approaches, GARP was detected in and around blood and lymphatic vessels, in (αSMA+) fibroblasts, and in perivascular cells associated with an abundant matrix. Strikingly, GARP was detected in LECs forming the subcapsular sinus and high endothelial venules (HEVs), two vascular structures localized at the interface between LNs and the afferent lymphatic and blood vessels. Altogether, we here provide the first distribution maps for GARP in human and murine LNs.

## 1. Introduction

Many types of cancer, such as breast, cervical, head and neck, and pancreatic carcinomas, as well as melanomas, are prone to disseminate through the lymphatic system [1,2,3,4,5,6]. Lymph nodes (LNs) are thus the first metastatic relay. The presence or absence of metastatic tumor cells in the sentinel LN, the first tumor-draining LN, is strongly associated with poor clinical outcomes and thus a crucial parameter for clinicians [7]. After colonizing LNs, metastatic tumor cells can seed distant organs and form systemic metastases [8,9]. The mechanisms underlying the cascade of events leading to LN metastases remain poorly understood.

Before nodal dissemination, the primary tumor modulates the microenvironment of its draining LN by secreting soluble factors (growth factors, cytokines) or releasing extracellular vesicles transported by lymphatic vessels [9,10,11,12,13]. This tissue remodeling occurs before the arrival of the first tumor cells. It leads to elaborating a so-called pre-metastatic niche permissive for subsequent metastatic cell survival and growth. The main features of this pre-metastatic LN niche include increased lymphangiogenesis and lymph flow, the recruitment of myeloid cells, and a reduction in effector lymphocyte numbers and function [11,14,15,16]. Strikingly, reported modifications of the immune landscape (changes in the proportion of CD8, Foxp3, CD20, or PD-1-expressing cells) suggest the elaboration of an immunosuppressive microenvironment in LNs [2]. In this context, an increased proportion of Foxp3+ regulatory T cells (Tregs) in LNs is expected to contribute to the suppression of anti-tumor immunity.

Upon activation of the T cell receptor, Tregs express a transmembrane protein called glycoprotein A repetitions predominant (GARP, encoded by the *LRRC32* gene). GARP covalently binds and presents latent transforming growth factor beta 1 (TGF-β1) on the Treg surface [17]. TGF-β1 is a pro-fibrotic and potently immunosuppressive cytokine that plays major roles in maintaining immune tolerance [18]. TGF-β1 is produced by virtually all cell types in a latent, inactive form (latent TGF-β1), in which the mature TGF-β1 dimer is non-covalently bound to the latency-associated peptide (LAP), preventing the binding of the cytokine to its receptor. To become active, TGF-β1 must be released from the LAP. In cells that do not express GARP, latent TGF-β1 is produced and secreted in association with latent TGF-β binding proteins (LTBPs), to which it associates via a disulfide linkage. Secreted LTBP:(latent)TGF-β1 complexes are deposited in the extracellular matrix, constituting a reservoir of latent TGF-β1 ready for activation by other cells. In cells that express GARP, in contrast, latent TGF-β1 associates preferentially with GARP via the formation of disulfide bonds, implicating the same LAP cysteine as that implicated in binding to LTBPs in other cell types [17]. GARP:(latent)TGF-β1 complexes are presented on the surface of human Tregs, where they can bind and be activated by integrin αVβ8 [17]. Monoclonal antibodies against GARP:TGF-β1 have been developed to block TGF-β1 activation and immunosuppression by GARP-expressing Tregs, but not by cells that do not express GARP [17,19,20]. They are currently tested for the immunotherapy of cancer in patients with locally advanced metastatic solid tumors [21] (clinicaltrials.gov NCT03821935 and NCT05822752).

Intriguingly, GARP is also expressed by other immune and non-immune cell types, including megakaryocytes and platelets [22], B cells [23], mesenchymal cells [24], and blood endothelial cells [21,25,26]. The spatial distribution of GARP-expressing non-immune cells in the LNs has not been studied previously. Non-immune cells contribute to the LN’s architecture, compartmentalization, and function in the anti-tumoral immune response [27].

Blood and lymphatic endothelial cells (BECs and LECs, respectively) constitute two vascular networks implicated in antigen and cell transport. Of note, high endothelial venules (HEVs) are specialized blood vessels expressing the peripheral node addressin (PNAd). They are involved in the recruitment of naive lymphocytes [9,28] in lymphoid tissues and also contribute to the egress of metastatic cells from the LN [8,9]. Fibroblastic reticular cells (FRCs) and LECs elaborate a three-dimensional reticular meshwork important for the transport of antigens and signaling molecules into the LN parenchyma, as well as for immune cell trafficking, priming and activation [27,29]. Recent advances in single-cell RNA sequencing have highlighted the heterogeneity of FRCs [30] and vascular cells (BECs, HEVs, and LECs), both in murine and human LNs [31,32,33,34]. Which of these cells express GARP in LNs is unclear.

Our study was carried out to identify and localize the different non-immune cell types expressing GARP and thus GARP:TGF-β1 complexes in non-metastatic and metastatic LNs. We here combined the analyses of publicly available scRNA-Seq datasets, immuno-histological stainings (IHCs), and in situ RNA hybridization approaches to map GARP expression in fibroblastic and vascular components of human and murine LNs. For the first time, we provide the spatial distribution of GARP expressing by perivascular, blood, and lymphatic endothelial cells in tumor-draining LNs.

## 2. Materials and Methods

### 2.1. Single-Cell RNA Sequencing of Human LN Cells

Human LN data (EGAD00001008311, European Genome-Phenome Archive database from Abe et al. were processed and analyzed using the R package Seurat (v.4.3.0) in RStudio (v.4.1.3) [31]. After removing ribosomal genes from cells that were apoptotic or lysed (more than 5% of mitochondrial genes), we filtered out genes expressed in fewer than three cells and cells expressing fewer than 200 unique features (low-quality cells). Cells with unique feature counts higher than 7500 genes (more than twice the median number, likely corresponding to doublets) were also removed. We then normalized data using the NormalizeData function and extracted highly variable features using the FindVariableFeatures function. Normalized data underwent a linear transformation (scaling, ScaleData function) and a principal component analysis (PCA) based on variable features using the RunPCA function. Graph-based clustering was then performed according to gene expression profiles using the FindNeighbors (dims = 1:50) and FindClusters (resolution: 0.8) functions. Results were visualized using a UMAP nonlinear dimensional reduction technique by running RunUMAP and DimPlot functions. The clustering of cells was annotated through the differentially expressed genes (DEGs) by running the FindAllMarkers function and the previous annotation described by Rodda et al. Cell clusters were illustrated using canonical cell type markers for lymphatic endothelial cells (LEC: *PROX1+*, *PDPN+*), blood endothelial cells (BEC: *CD34+*, *PECAM1+*), mesenchymal stromal cells (SCs: *COL1A1+*, *PDGFRB+*) expressing PTX3 (SCs-PXT3), SCs expressing C7 (SCs-C7), SCs expressing SFRP4 (SCs-SFRP4), SCs expressing AGT (SCs-AGT), adventitial/medullary reticular cells (ACs-MedRCs: *PCOLCE2+*, *MFAP5+*, *IGFBP6+*), and perivascular cells (PvCs: *MYL9+*, *ITGA7+*, and *ACTA2+*).

### 2.2. Single-Cell RNA Sequencing of Mouse LN Cells

Murine LN pre-processed data (GEO GSE202068 [35]) were processed and analyzed using the R package Seurat (v.4.3.0) in RStudio (v.4.1.3). After removing ribosomal genes from cells that were apoptotic or lysed (more than 5% of mitochondrial genes), we filtered out genes expressed in fewer than three cells and cells expressing fewer than 200 unique features (low-quality cells). Cells with unique feature counts higher than 7500 genes (more than twice the median number, likely corresponding to doublets) were also removed. We then normalized data using the NormalizeData function and extracted highly variable features using the FindVariableFeatures function. Normalized data underwent a linear transformation (scaling, ScaleData function) and a principal component analysis (PCA) based on variable features using the RunPCA function. Graph-based clustering was then performed according to gene expression profiles using the FindNeighbors (dims = 1:20) and FindClusters (resolution: 0.4) functions. Results were visualized using a nonlinear dimensional reduction UMAP technique by running RunUMAP and DimPlot functions. The clustering of cells was annotated through the differentially expressed genes (DEGs) by running the FindAllMarkers function and the previous annotation described by Rodda et al. Cell clusters were illustrated using canonical cell type markers for lymphatic endothelial cells (LEC; Prox1, Flt4, and Lyve1), subdivided into two clusters LEC I (*Ackr4*, *Foxc2*, and *Cdln11*) and LEC II (*Madcam* and *Bmp2*), blood endothelial cells (BEC: *Pecam1* and *Cdh5*), marginal reticular cells (MRCs: *Enpp2* and *Cxcl13*), T cell reticular cells (TRCs: *Ccl19* and *Ccl21a*), adventitia cells (Acs: *Igfbp6* and *Mfap5*), medullary reticular cells (MedRCs: *Penk* and *Tmeff2*), and perivascular cells (PvCs: *Myl9*, *Notch3*, and *Acta2*).

### 2.3. In Vitro Cell Culture

We used primary human dermal lymphatic microvascular endothelial cells (HMVEC-dLyAd, CC-2810, Lonza, Verviers, Belgium) and human umbilical vein endothelial cells (CC-2519A, Lonza, Verviers, Belgium), herein referred to as LECs and HUVEC, respectively. These cells were cultured as a monolayer in EGM2-MV medium (complete medium) (CC-3202 for LECs and CC-3162 for HUVEC, Lonza, Verviers, Belgium) until confluence. Primary human lymphatic fibroblasts (HLFs from ScienCell, #2530, Carlsbad, CA, USA) were cultured in complete fibroblast medium (FM, Cat. #2301, ScienCell) until confluence was achieved, according to the manufacturer’s instructions. Jurkat cells (clone E6-1) were obtained from the American Type Culture Collection (Manassas, VA, USA). As previously described, Clone E6-1 was transduced with a lentivirus encoding GARP to generate neomycin-resistant Jurkat + GARP cells [36]. Jurkat + GARP cells were cultured in RPMI medium (Gibco) supplemented with 10% fetal bovine serum (10270-106, Thermofisher, Waltham, MA, USA), 1% glutamine (25030-123, Thermofisher), and 1% penicillin–streptomycin (15140-122, Thermofisher), in the presence of neomycin (1 mg/mL) (N6386, Sigma-Aldrich, Darmstadt, Germany).

### 2.4. Western Blot

Cells were lysed with cell lysis buffer 1x composed of 20 mM Tris-HCl pH 7.5, 150 mM NaCl, 1 mM Na2EDTA, 1 mM EGTA, 1% Triton, 2.5 mM sodium pyrophosphate, 1 mM β-glycerophosphate, 1 mM Na3VO4, 1 µg/mL leupeptin (#9803, Cell Signaling, Danvers, MA, USA), and 1× protease/phosphatase inhibitor cocktail (Complete and phosSTOP, Roche, Indianapolis, IN, USA). Lysate samples were separated on acrylamide gels (10%) in a reducing condition with SDS at 20 µg/well and then transferred onto PVDF transfer membranes (88518, ThermoScientific, Waltham, MA, USA). Membranes were probed by overnight incubation at 4 °C with the indicated antibodies followed by 1 h incubation at room temperature with horseradish peroxidase-coupled secondary antibody (7076, Cell Signaling, Danvers, MA, USA) and enhanced chemiluminescent substrate (NEL104001EA, PerkinElmer, Waltham, MA, USA) using an Amersham ImageQuant 800 (GE Healthcare, Chicago, IL, USA). The following antibodies were used: GARP (1/1000; LRRC32 monoclonal antibody, Plato-1, ALX-804-867-C100, Enzo Life Sciences, Farmingdale, NY, USA) and GAPDH (1/10,000; MAB 374, Millipore, Burlington, MA, USA).

### 2.5. Flow Cytometry

Cell suspensions were harvested using Accutase (#07922, Stemcell Technologies, Vancouver, BC, Canada) at 37 °C for 5 to 10 min. The single-cell suspensions obtained were counted and immediately stained with antibodies against the following surface markers: GARP PE (clone Plato-1, Enzo Life Sciences, Farmingdale, NY, USA), integrin αV (clone NKI-M9; CD61), integrin β3 (clone VI-PL2) (Biolegend, San Diego, CA, USA), integrin β6 (clone #437211), integrin β8 (clone #416922, R&D Systems, Minneapolis, MN, USA) in the presence of a viability dye (Invitrogen, Waltham, MA, USA) and anti-CD16/32 to block FcgRs (Biolegend). Analyses were performed on an FACSCanto™ II flow cytometer (DIVA, BD Biosciences, San Jose, CA, USA), and data were computed using the FlowJo software vX.0.7 (Tree Star, Ashland, OR, USA).

### 2.6. Multiplexed Immunofluorescence on Human LN Sections

Human LN samples from breast or cervical cancer patients were obtained from CHU ULiège Biobank. Seven µm thick cryosections from human LNs were mounted on a Superfrost microscope slide and fixed in formaldehyde 4% for 5 min, washed with demineralized water (ddH_2_O) and phosphate-buffered saline (PBS), and then incubated with 3% H_2_O_2_ (8070-4, Carl Roth, Karlsruhe, Germany) to block endogenous peroxidases. Sections were blocked with animal-free blocking solution (15019 L, Cell Signaling), followed by an incubation with either 5 µg/mL of mouse monoclonal anti-human GARP antibody (clone MHG-6 [20]) in Dako antibody diluent or no primary antibody (negative control) for 90 min. After two washes with PBS supplemented with Tween20 (PBS-T), the EnVision-HRP secondary antibody (K4001, Dako Agilent, Diegem, Belgium) was incubated at room temperature for 30 min. Staining was amplified using tyramide signal amplification working solution (TSA, NEL741001KT, PerkinElmer) containing fluorescein isothiocyanate dye (1:500, FITC) for 10 min, washed thrice with PBS-T. The following antibodies were used in combination with GARP (following the same steps as above): rabbit monoclonal anti-FoxP3 antibody (clone EPR15038-69, Abcam, Cambridge, UK) (1:200), mouse monoclonal anti-CD34 antibody (clone Qbend 10, Abcam) (1:200), sheep polyclonal anti-PDPN (AF3670, R&D Systems) (1:200), monoclonal rat anti-PNAd Alexa Fluor 488 (High Endothelial Venule Marker, clone MECA79, eBioscience, San Diego, CA, USA) (1:100), αSMA (ab5694, Abcam) (1:200), and CD31 (ab24590, Abcam) (1:200). Ready-to-use EnVision+ System-HRP Labelled Polymer anti-Rabbit (K4003, Dako), anti-mouse IgG1 coupled with HRP (115-035-205, Jackson ImmunoResearch, Cambridge, United Kingdom) (1:1000), and anti-sheep coupled with HRP antibody (1:3000) were used as secondary antibodies, followed by TSA incubation with Cy3 or Cy5 (1:2000) instead of fluorescein. For the dual immunostaining GARP/HEV (PNAd), amplification of GARP with TSA was made with Cy3. A last wash with PBS-T was made before mounting the slides with Fluoromount containing DAPI (0100-20, SouthernBiotech, Birmingham, AL, USA).

### 2.7. Ear Sponge Assay

C57Bl6 female mice (6 to 8 weeks old) were used throughout this study. The animals were maintained under a 12 h light–dark cycle with free access to food and water. Gelatin sponges were incubated with tumor cells (2 × 10^5^ B16F10 cells/sponge) or control medium (serum-free DMEM without tumor cells) for 30 min in serum-free-DMEM, embedded with collagen, and implanted into mouse ears as previously described [32,33]. Bioluminescence was detected in animals bearing ear sponges soaked with luciferase-expressing cells using the in vivo Imaging System IVIS 200 (Xenogen Corp.; Alameda, CA, USA). At the end of the experiment, the sponges and cervical LNs were harvested, incubated in 4% formol (11699408, VWR, Leuven, Belgium) for 16 h, dehydrated in ethanol, and fixed in paraffin (X881.2, Leica, Frankfurt, Germany).

### 2.8. In Situ RNA Hybridization

The mRNA in situ hybridization of *Lrrc32* (Garp) and *Prox1* was measured on mice LN tissue sections using the RNAscope assay according to the manufacturer’s instructions (Advanced Cell Diagnostics, Bioké, Leiden, The Netherlands). Tissue sections (10 μm) were deparaffinized/rehydrated and hybridized with Mm-Lrrc32-C1 (#592941-C1, Bioké, Leiden, The Netherlands) probes and/or Mm-Prox1-C2 (#488591-C2, Bioké, Leiden, The Netherlands) with an RNAscope 3-plex negative control probe (#320871, Bioké, Leiden, The Netherlands). The hybridization signal was amplified with RNAscope Multiplex Fluorescent reagent kit V2 (#323135, Bioké, Leiden, The Netherlands) and with OPAL (Opal 520, PN FP1487001KT; Opal 570, PN FP1488001KT; Opal 620, PN FP1495001KT).

After hybridization, immunostaining was performed with one of the following antibodies incubated overnight at 4 °C: Alexa Fluor 488-PNAd (MECA-79, eBioscience), FoxP3 (ab191416, Abcam), Lyve-1 (AF2125, R&D Systems), CD31 (ab28364, Abcam), or αSMA (ab5694, Abcam).

### 2.9. Slide Scanning and Image Analysis with Olyvia and QuPath

For human samples, digital 3 or 4-color images of the stained tissue sections were digitalized using a NanoZoomer 2.0-HT system (Hamamatsu Photonics, Hamamatsu, Japan) at 20× magnification with a resolution of 0.23 µm/pixel. Mice samples of digital 3 or 4-color images were acquired with the SLIDEVIEW VS200 research slide scanner (Olympus, Anvers, Belgium) equipped with a UPlan-XApo 20× 0.8× objective (Olympus) and with a Hamamatsu ORCA-Flash camera, using DAPI, FITC, Cy3, and Cy5 filter sets. Some high-magnification images were generated with a confocal Zeiss LSM880 Airyscan microscope (Zeiss, Oberkochen, Germany) and a 40× or 63× objective lens. The relative quantity of GARP immunostaining in human LN samples was quantified with QuPath software (v0.4.3, [37]) using a deep learning model composed of 6 layers of different nodes (8, 10, 10, 10, 10, and 10 nodes) with the library OpenCV (module ann_mlp) and the Gaussian Laplacian features with an output of classification method.

### 2.10. Statistics

Statistical analyses were performed with GraphPad Prism 9.0 software using the Mann–Whitney test or one-way ANOVA, two-tailed as indicated in the figure legends. Data are shown as mean ± SD, and differences were considered statistically significant when *p* < 0.05, as indicated by asterisks with *p* < 0.05 (*), *p* < 0.01 (**), and *p* < 0.001 (***).

### 2.11. Study Approval

Animal experiments complied with the Animal Ethical rules of the University of Liège (Liège, Belgium) after approval from the local Animal Ethical Committee. Human LN samples were stored in the biobanks of the University of Liège (CHU, Liège, Belgium) after study approval by local ethics committees. The use of human body material from the biobank does not require consent forms under the Belgian law of 19 December 2008.

## 3. Results

### 3.1. Single-Cell RNA Sequencing Analysis of Human LNs Uncovers the LRRC32 Gene Expression by Subpopulations of Endothelial and Perivascular Cells in Human LNs

**Figure 1:**
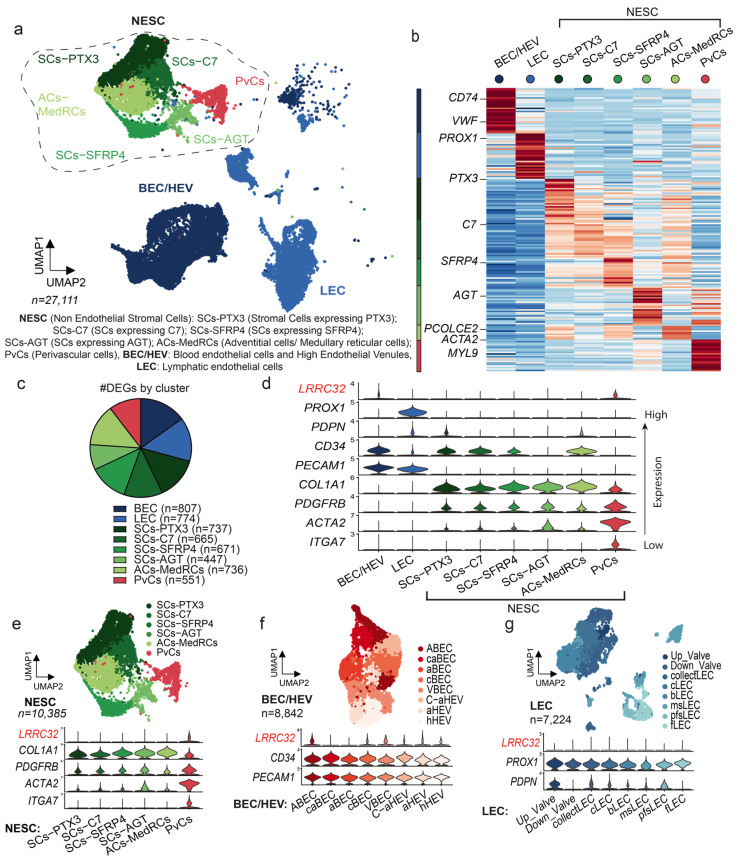
LRRC32 gene expression analysis in individual cells derived from metastasis-free human LNs. (**a**) UMAP plot clusters 27,111 cells from 9 metastasis-free LNs into 8 distinct groups (BECs/HEVs, LECs, SCs-PTX3, SCs-C7, SCs-SFRP4, SCs-AGT, ACs-MedRCs, and PvCs). (**b**) Heatmap showing the expression levels of the top-ranking marker genes in each cluster. Key genes are indicated on the left. (**c**) Number of DEGs in each cluster (**d**). Violin plot showing expression of genes of interest including *LRRC32* (in red) in each cluster. (**e**–**g**) UMAP plot clusters (**e**) non-endothelial stromal cells (NESCs), (**f**) BECs/HEVs, and (**g**) LECs, and violin plots showing expression of genes of interest, including *LRRC32* (in red) in each cluster.

The LN, the main organ in which immune response takes place, comprises immune and non-immune cells, such as BECs, LECs, and FRCs, as well as other mesenchymal cells. In order to identify which non-immune cells express *LRRC32* (the gene encoding GARP) in human LNs, we started our analysis by examining available single-cell RNA sequencing (scRNA-Seq) datasets from human LN samples [31] (Figure 1a). In one of the datasets [31], non-sentinel non-enlarged LNs were taken from patients with a neoplasm (*n* = 9) and their malignancy-freeness was verified with a pan-cytokeratin marker. LN CD45- cells were separated into non-endothelial stromal cells (NESCs), BECs/HEVs, and LECs. Different subclasses were clustered through differentially expressed genes (DEGs) to highlight their specificity of expression and functionality (Figure 1b,c). Cells were classified into eight groups (Figure 1a–c): blood endothelial cells (BECs: *PECAM1+*, *CD34+*), lymphatic endothelial cells (LECs: *PROX1+*, *PDPN+*, *PECAM1+*), mesenchymal stromal cells (SCs: *COL1A1+*, *PDGFRB+*) expressing PTX3 (SCs-PXT3), SCs expressing C7 (SCs-C7), SCs expressing SFRP4 (SCs-SFRP4), SCs expressing AGT (SCs-AGT), adventitial/medullary reticular cells (ACs-MedRCs: *PCOLCE2+*, *MFAP5+*, *IGFBP6+*), and perivascular cells (PvCs: *ACTA2+*, *MYL9+*, *ITGA7+*). The violin plot provides an insightful visualization of the scRNA-Seq expression profiles of the *LRRC32* gene within the different subclasses of stromal cells (NESCs, BECs/HEVs, LECs, and PvCs) (Figure 1d). Of note, *LRRC32* was expressed by PvCs and BECs/HEVs, and was detected at a very low level in NESCs and LECs (Figure 1d). A focus on PvCs and NESCs confirmed a higher *LRRC32* expression level in PvCs as compared with NESCs (Figure 1e). Interestingly, *LRRC32* expression was detected in almost all BEC/HEV subtypes: large arteries (ABECs), arteries surrounding the LN capsule (caBECs), arterioles (aBECs), capillary BECs (cBECs), transitional BECs between capillary BECs and activated HEVs (C-aHEVs), large veins (VBECs), activated HEVs (aHEVs), and homeostatic HEVs (hHEVs) (Figure 1f). In sharp contrast, *LRRC32* was not or only faintly expressed in the different LEC subtypes (Figure 1g). Collectively, these data suggest a role of GARP in different subpopulations of human blood endothelial cells and perivascular cells [38].

### 3.2. Expression of GARP and Integrins Are Produced In Vitro by Human Endothelial Cells and LN Fibroblasts

**Figure 2:**
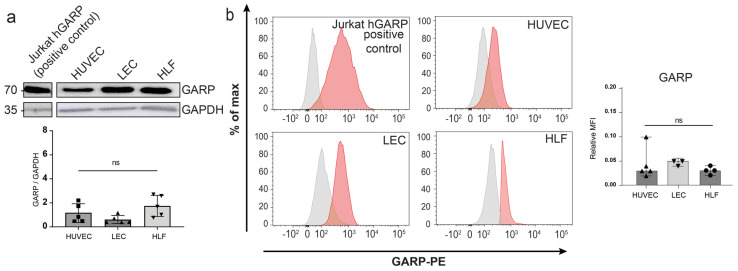
Evaluation of GARP in HUVEC, LEC, and HLF cells cultured under basal conditions. (**a**) Western blot analysis of GARP expression. The blot is a representative blot out of 4 independent experiments. The bar graph shows the quantification of GARP protein levels relative to the GAPDH protein signal (GARP/GAPDH signals) (*n* = 4, means ± SD, n.s. determined by one-way ANOVA). (**b**) Flow cytometry analysis of GARP at the surface of primary cells. Jurkat cells overexpressing GARP (Jurkat−hGARP) were used as a positive control. The isotype control is represented in grey, and the positive signal is depicted in red as a percentage of the maximum. The relative MFI of GARP in flow cytometry is represented with a bar graph (*n* ≥ 3, means ± SD, n.s., no significance, determined by one-way ANOVA).

**Figure A1:**
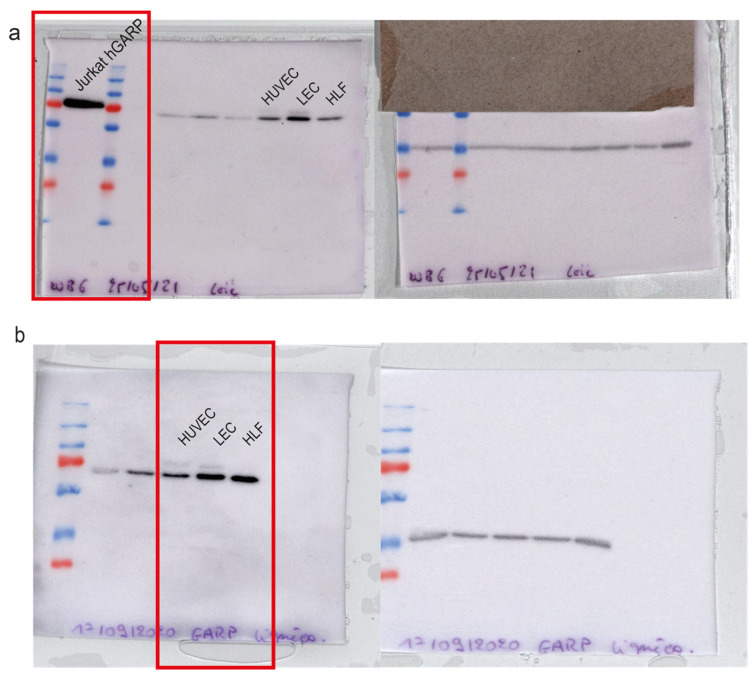
Full scans of Western blot images shown in Figure 2a. (**a**) Western blot membrane of GARP in Jurkat hGARP used as a positive control. The red rectangle corresponds to the first lane in Figure 2a. (**b**) Western blot membrane of GARP on HUVECs, LECs, and HLFs in which the red rectangle corresponds to the right part of Western blot shown in Figure 2a.

**Figure A2:**
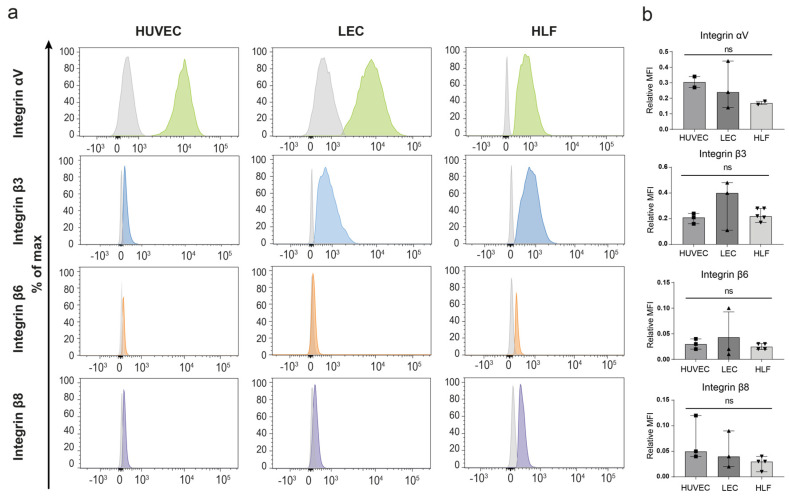
Evaluation of GARP integrins partners in HUVEC, LEC, and HLF cells cultured in basal condition. (**a**) Flow cytometric analyses were conducted to evaluate the expression of αV (green), β3 (blue), β6 (orange), and β8 (purple) integrins in the different cells. The isotype control is depicted in grey, and positive signals for each integrin are shown in color (described above) as a percentage of the maximum. (**b**) The relative MFI is represented with a bar graph for each integrin shown on the left panel; n is at least equal to 4 (n ≥ 4, means ± SD, n.s, no significance, determined by one−way ANOVA).

We next assessed GARP protein expression in various primary cultures, including human umbilical vein endothelial cells (HUVECs), human LECs, and human lymphatic fibroblasts (HLFs) in basal conditions. Jurkat cells transduced to express the human GARP protein (h-GARP) were used as a positive control. Western blot analyses revealed similar levels of GARP expression in the different primary cells (Figure 2a and Figure A1). Flow cytometry analyses under non-permeabilizing conditions further validated these findings and confirmed the presence of GARP on the cell surface of HUVECs, LECs, and HLFs (Figure 2b). Given the potential contribution of integrins αVβ6 and αVβ8 to the activation of latent TGF-β1 presented by GARP on the cell surface [17], we examined the presence of these integrins at the surface of the different cell types, as well as that of the more common integrin αVβ3. Flow cytometry detected almost similar and important levels of αV and β3 subunits in the three primary cell cultures (Figure A2). The β6 and β8 integrin subunits were also detected in HUVECs, LECs, and HLFs. These results established that, in basal culture conditions, protein GARP, αVβ6, and αVβ8 are co-expressed at the surface of primary HUVEC, LEC, and HLF cultures.

### 3.3. Detection and Mapping of GARP in Human Metastatic LN Samples

**Figure 3:**
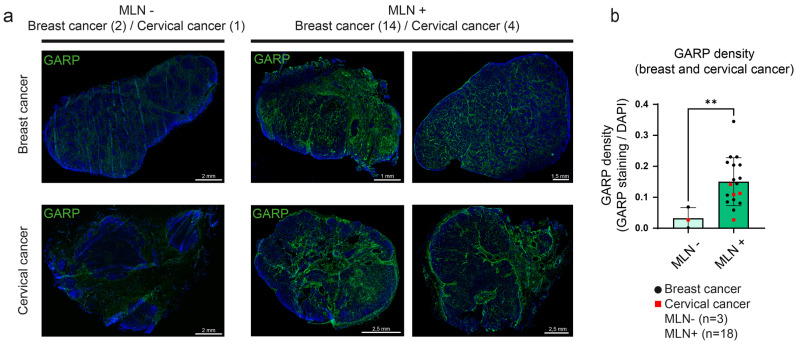
Multiplex immunofluorescence identifies GARP expression in human LN. (**a**) Immunofluorescence was conducted on frozen sections of human LN derived from metastatic-negative (MLN−, *n* = 3) and metastatic-positive (MLN+, *n* = 18) LNs from patients diagnosed with breast cancer (BC; 3 MLN− and 14 MLN+) or cervical cancer (CC; 1 MLN− and 4 MLN+). The sections were stained with anti-GARP antibody (in green) and DAPI for nuclei (in blue). Scale bar = 2, 1, 1.5, or 2.5 mm (**b**) Computer-assisted quantification of GARP density using QuPath (relative density with DAPI area) in MLN− and MLN+. The bar graph is represented with individual data points, and results are expressed by mean ± SD (** *p* = 0.0053 determined by the Mann–Whitney test).

We next investigated the spatial distribution of GARP-expressing cells and GARP-positive areas in LNs of patients with breast (BC, *n* = 14) or cervical (CC, *n* = 4) cancer. Immunohistochemical analyses were conducted on non-metastatic (MLN−) (total *n* = 3) and metastatic LNs (MLN+) (*n* = 18). It is worth noting that the anti-GARP antibody (clone MHG-6) can only be used on frozen tissue sections, which limited the number of (normal and metastatic) human tissue samples amenable to analyses. Interestingly, the density of GARP staining was higher in MLN+ than in MLN− (Figure 3a). Despite the reduced number of MLN− samples, we noticed an approximative five-fold increased density of GARP staining in MLN+ compared to MLN− (** *p* = 0.0053) (Figure 3b). As expected, we found GARP+/FoxP3+ cells, corresponding to activated Tregs, and GARP-/FoxP3+ cells, corresponding mostly to non-activated Tregs, in both MLN+ and MLN− samples (Figure S1a–c). Areas with strong GARP staining but without FoxP3 staining were found in both types of samples, indicating the presence of non-Treg GARP-expressing cells (Figure S1a–c). Most GARP+FOXP3- areas were found around vessels and in the extracellular matrix. This observation prompted us to postulate that peri-vascular and vascular cells express GARP in LNs (Figure S1b,c).

**Figure 4:**
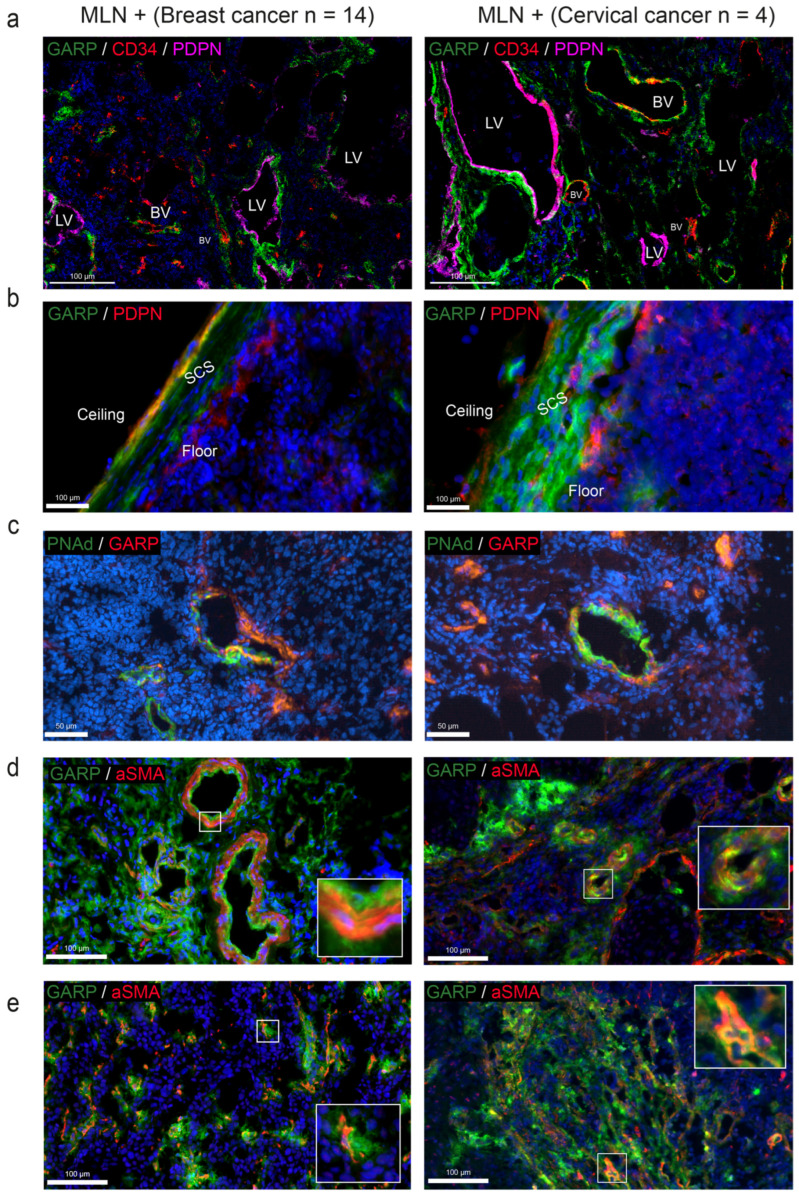
Multiplex immunofluorescence identifies GARP expression in lymphatic and blood vessels in human LNs. (**a**) Multiplex immunofluorescence staining of GARP (in green), CD34 (in red), podoplanin (in pink), and nuclei (DAPI, in blue) on MLN+ from patients with breast cancer (*n* = 14) or with cervical cancer (*n* = 4). LV: lymphatic vessel, BV: blood vessel. Scale bar = 100 µm. (**b**) Multiplex immunofluorescence staining of GARP (in green), PDPN (in red), and nuclei (DAPI, in blue) focused on the LN capsule on MLN+ from patients with breast cancer (*n* = 14) or with cervical cancer (*n* = 4). SCS: subcapsular sinus. Scale bar = 100 µm. (**c**) Multiplex immunofluorescence staining of GARP (in red), PNAd (in green), and nuclei (DAPI, in blue) on MLN+ from patients with breast cancer (*n* = 14) or with cervical cancer (*n* = 4). Scale bar = 50 µm. (**d**) Multiplex immunofluorescence staining of GARP (in green), αSMA (in red), and nuclei (DAPI, in blue) of MLN+ from patients with breast cancer (*n* = 14) or with cervical cancer (*n* = 4) or (**e**) in the ECM. Scale bar = 100 µm.

To investigate GARP protein expression in the blood and lymphatic LN vasculatures, a triple co-staining of GARP, CD34 (a marker of blood vessels), and podoplanin (PDPN, a marker of lymphatic vessels) was performed and analyzed by confocal microscopy (Figure 4a). GARP staining was detected in the cell wall of CD34+/PDPN− blood vessels and around CD34-/PDPN+ lymphatic vessels. (Figure 4a). We also detected GARP staining in the subcapsular sinus (SCS), both in the layer directly in contact with the LN parenchyma (floor LECs) and in the external layer (ceiling LECs) (Figure 4b). In line with the detection of *LRRC32* expression in HEVs in scRNA-Seq data, GARP positivity was also found in PNAd+ HEV vessels (Figure 4c). Given the scRNA-Seq analysis identifying perivascular cells as a putative cellular source of GARP, we next focused on those cells identified by *ACTA2* (αSMA) expression. IHC with an anti-αSMA antibody confirmed the presence of GARP+/αSMA+ cells surrounding blood vessels or isolated in the ECM, likely corresponding to fibroblastic cells (Figure 4d,e). In contrast to the scRNAseq data suggesting *Itga7* expression by perivascular cells, only a few ITGA7+ cells were noted, in contrast to a widespread expression of αSMA (Figure A4). Collectively, our analyses lead to the mapping of GARP in human LNs (Figure 4a–e), revealing the presence of GARP in blood vessels, HEVs, and the SCSs, as well as αSMA+ perivascular and fibroblastic cells.

### 3.4. Single-Cell RNAseq Analysis Uncovers the Lrrc32 Gene Expression in Endothelial and Peri-Vascular Cells of Murine LNs

**Figure 5:**
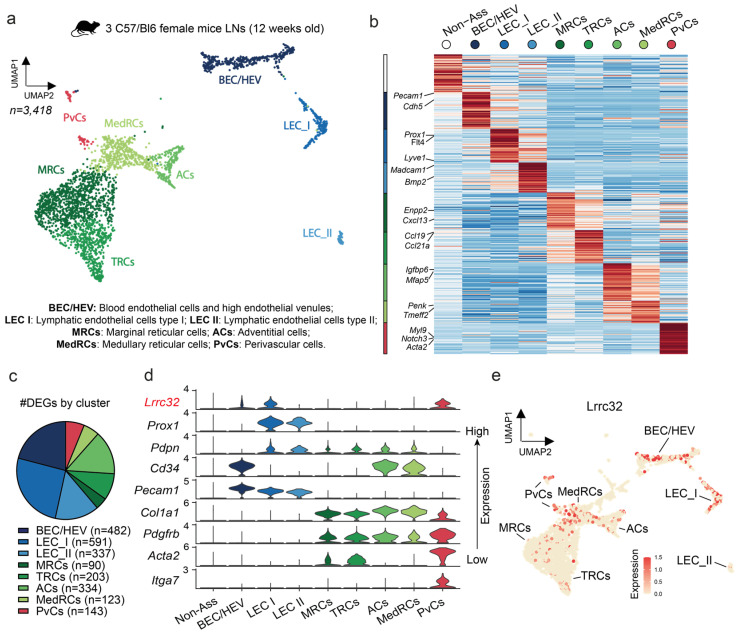
Lrrc32 gene expression analysis in individual cells derived from metastasis-free mice LNs. (**a**) UMAP plot clusters 3,418 cells from 3 C57/Bl6 female mice LNs cells into 8 distinct groups (BEC/HEV, LEC I, LEC II, MedRCs, ACs, MRCs, TRCs, and PvCs). (**b**) Heatmap showing the expression levels of the top-ranking marker genes in each cluster. Key genes are indicated on the left. (**c**) Number of DEGs in each cluster. (**d**) Violin plot showing expression of genes of interest including Lrrc32 (in red) in each cluster. (**e**) UMAP plot of Lrrc32 expression level in each cluster.

We next analyzed murine LN datasets of scRNA-Seq (Figure 5a,b) and focused our interest on FRCs (*Col1a1+*, *Pdgfrb+*), BECs/HEVs (*Pecam1+*, *Cd34+*), BECs/HEVs, and LECs (*Prox1+*, *Pdpn+*, *Pecam1+*). In each cell type, subclusters co-exist, characterized by distinct gene expression profiles related to their specific functions [31]. The exploration of the previously published dataset of naive mice LNs revealed different FRC subtypes: (i) marginal reticular cells (MRCs; *Enpp2+*, *Cxcl13+*) dispersed at the basis of the SCS, which are supposed to play a role in barrier defense; (ii) T cell reticular cells located near lymphocyte follicles (TRCs; *Ccl19+*, *Ccl21a+*); (iii) medullary reticular cells (MedRCs; *Inmt+*, *Penk+*, *Tmeff2+*), which are niche-restricted in the medullary sinus; (iv) adventitial cells in the medullary sinus (ACs; *Mfap5+*, *Igfbp6+*), which may support large vessels and secrete pro-(lymph)angiogenic factors; and (v) perivascular cells (PvCs; *Notch3+*, *Myl9+*, *Acta2+*, *Itga7+*) which are in the periphery of large blood vessels and have multiple functions of blood vessel support (Figure 5a,b) [30,35]. In this dataset, LECs were separated into LEC I (*Flt4+*, *Foxc2+*, *Ackr4+*, *Cldn11+*), which correspond to valve, collector, and ceiling lymphatic vessels, and LEC II (*Lyve1+*, *Madcam+*, *Bmp2+*), which are localized in the medullar and floor of the LN sinus. These different subclasses were clustered through differentially expressed genes (DEGs) to highlight their specificity of expression and functionality (Figure 5c). In line with the human data, *Lrrc32* mRNA expression was detected in BECs/HEVs and PvCs (Figure 5e). Furthermore, a LEC subpopulation, namely LEC I, corresponding mainly to collector lymphatic vessels and ceiling LECs, expressed *Lrrc32* at levels almost similar to that in PvCs (Figure 5d). These data indicate that GARP is expressed in murine BECs and PvCs, and a subpopulation of murine LECs [38].

### 3.5. Mapping of Lrrc32 mRNA Expression in Mouse LNs

**Figure 6:**
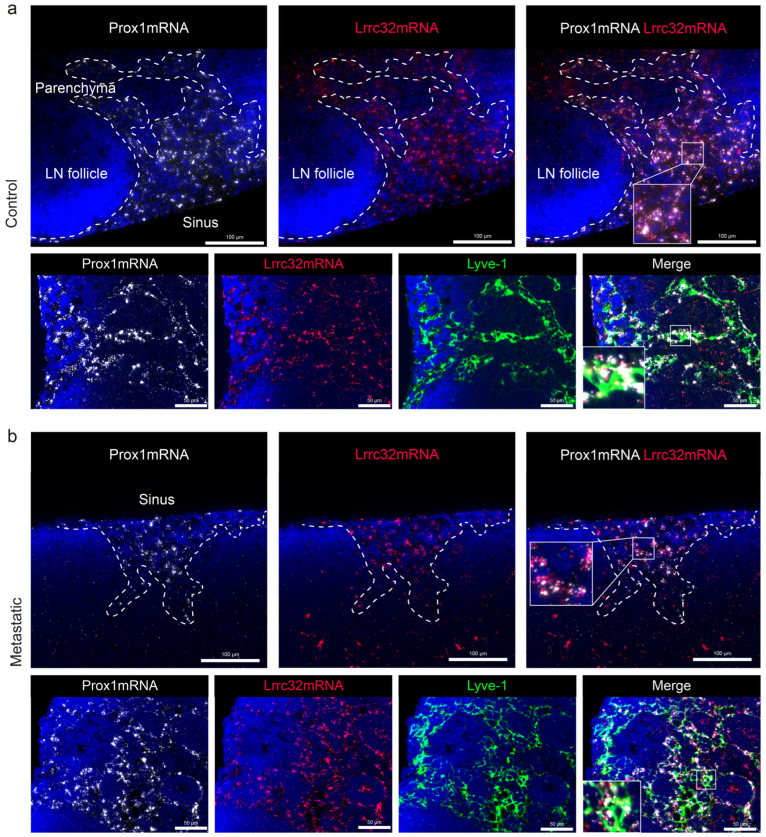
Mapping of *Lrrc32* mRNA (encoding Garp) in mouse LN parenchyma with hybridization. (**a**) mRNA detection by RNAscope of *Prox1* mRNA (white), *Lrrc32* mRNA (red), and/or coupled with Lyve-1 immunostaining on mouse cervical LN with a focus on the parenchyma area in control condition; (**b**) mRNA detection by RNAscope of *Prox1* mRNA (white), *Lrrc32* mRNA (red), and/or coupled with Lyve-1 immunostaining in a metastatic LN 3 weeks after B16F10 transplantation. Scale bar = 100 and 50 µm.

**Figure 7:**
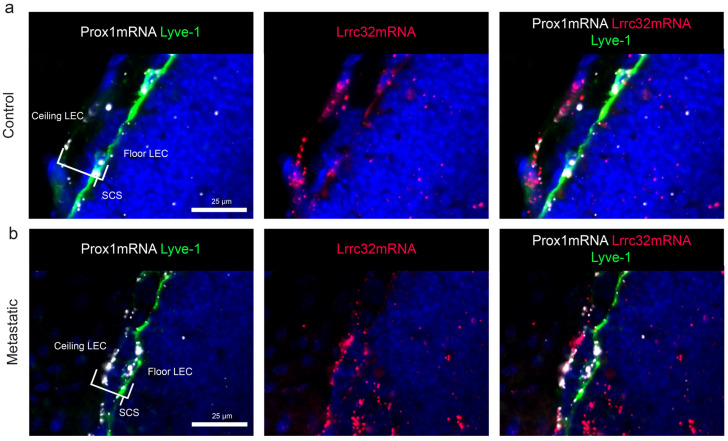
Mapping of *Lrrc32* mRNA (encoding Garp) in mouse LN SCS with hybridization. (**a**) mRNA detection by RNAscope of *Prox1* mRNA (white), *Lrrc32* mRNA (red) coupled with Lyve-1 immunostaining on mouse cervical LN with a focus on the SCS area in the control condition and (**b**) metastatic LN 3 weeks after B16F10 transplantation. Scale bar = 25 µm.

**Figure 8:**
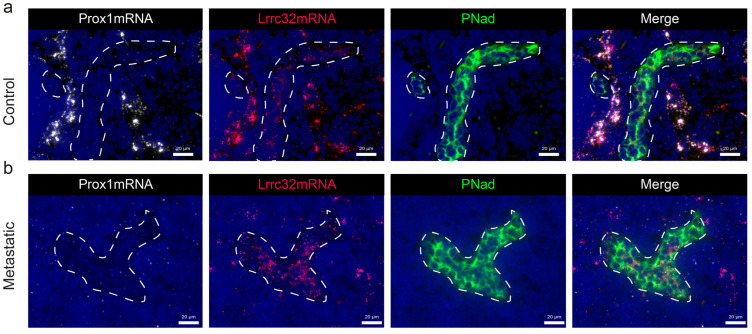
*Lrrc32* (encoding Garp) mRNA is expressed in HEV in mouse LN detected by hybridization. (**a**) mRNA detection by RNAscope of *Prox1* mRNA (white), *Lrrc32* mRNA (red) coupled with PNAd (HEV, in green) immunostaining on mouse cervical LN in control and (**b**) a metastatic LN 3 weeks after B16F10 transplantation conditions. The dashed line highlights HEV vessel sections. Scale bar = 20 µm.

**Figure 9:**
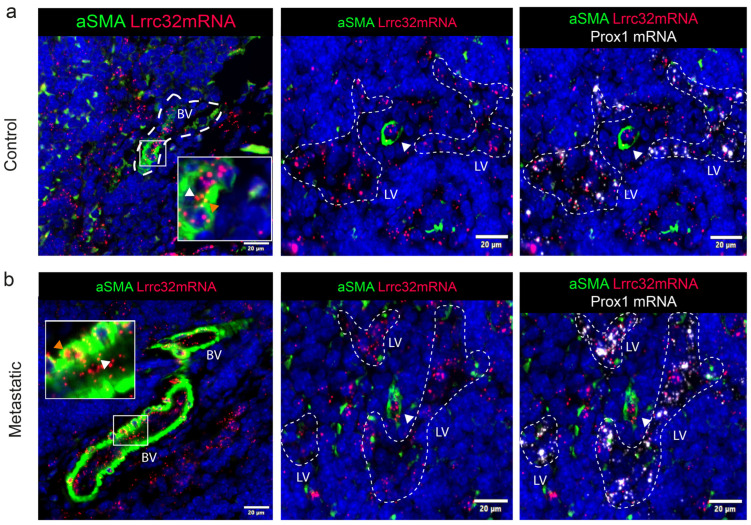
*Lrrc32* (encoding Garp) is expressed in blood and lymphatic vessels in mouse LN detected by hybridization. (**a**) mRNA detection by RNAscope of *Prox1* mRNA (white), *Lrrc32* mRNA (red) coupled with αSMA (in red), and CD31 (in pink) immunostaining on mouse cervical LN in control and (**b**) a metastatic LN 3 weeks after B16F10 transplantation conditions. The dashed line highlights the lymphatic vessel network. LV: lymphatic vessel, BV: blood vessel. White arrowheads show blood endothelial cells inside the vessel, and orange arrowheads indicate perivascular cells (PvCs). Scale bar = 20 µm.

**Figure A3:**
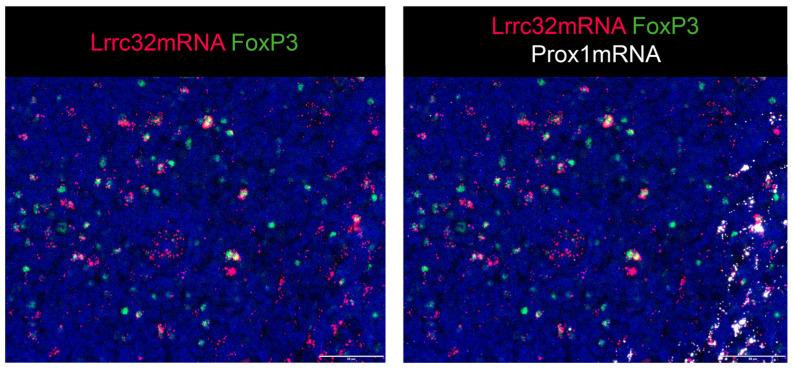
Lrrc32mRNA (Garp) and Prox1mRNA expression by RNAscope coupled with FoxP3 immunostaining as a control in Tregs cells. Scale bar = 50 µm.

**Figure A4:**
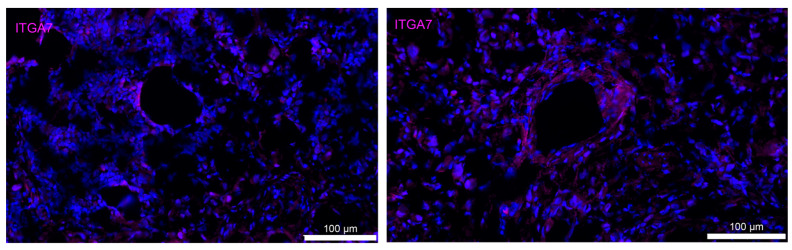
Immunostaining ITGA7 (pink) in human LN samples. Scale bar = 100 µm.

To determine the spatial distribution of *Lrrc32* expression in murine LNs, we used the pre-clinical ear sponge assay to induce LN metastasis [39]. In this model, gelatin sponges soaked with melanoma B16F10 melanoma cells were transplanted into the ears of mice. This leads to the formation of a local tumor and dissemination of metastatic tumor cells to the draining LNs 3 weeks post-implantation. Histological analyses confirmed the presence of metastases. Given the absence of suitable anti-murine GARP antibodies, we used the RNAscope technique to localize the *Lrrc32* (*Garp*) mRNA. First, we observe FOXP3+ cells surrounded by *Lrrc32* mRNA, used as a positive control (Figure A3). We concomitantly used a *Prox1* probe (a transcription factor expressed by lymphatic cells) and Lyve1 immunostaining to localize LECs. In the parenchyma of the LN, the *Lrrc32* mRNAs were found in *Prox1*+ lymphatic vessels in both control and metastatic LNs (Figure 6a,b). Lyve-1 staining confirmed *Lrrc32* mRNA expression in lymphatic vessels (Figure 6a,b). We next focused on the SCS, in which floor LECs (fLECs) are Lyve1+ while ceiling LECs (cLECs) are negative for Lyve-1. Lrrc32 mRNAs were detected in *Prox1*+/Lyve1+ cells (fLEcs) in contact with the parenchyma, as well as in external *Prox1*+/Lyve1- cells (fLECs) (Figure 7a,b). Interestingly, we also observed *Lrrc32* mRNA within PNAd+ HEVs (Figure 8a,b). Furthermore, the immunostaining of CD31 and αSMA indicated Lrrc32 expression in αSMA+ perivascular cells surrounding both blood (*Prox1−*/CD31+) and lymphatic vessels (*Prox1+*/CD31+). We also detected *Lrrc32+*/αSMA+ fibroblastic cells. A similar staining of blood endothelial cells, perivascular cells, and fibroblasts was detected in both control and metastatic mice LN samples (Figure 9a,b).

We thus mapped the expression of *Lrrc32* mRNA in mouse LNs (Figure 5 and Figure 8) and found *Lrrc32* expression in the lymphatic network (*Prox1*+/Lyve1+) of the SCS (including the ceiling and floor) and the parenchymal areas, as well as in perivascular αSMA+ cells around blood and lymphatic vessels.

## 4. Discussion

The originality of our study relies on investigating *LRRC32* mRNA and GARP protein expression in non-Treg cells in LN metastases, rather than in primary tumors. GARP was reported to mediate TGF-β1 activation and immunosuppression by Tregs in tumor-bearing mice, as blocking antibodies against GARP:TGF-β1 complexes induced regressions of tumors otherwise resistant to anti-PD-1 immunotherapy [40]. A Foxp3–GARP–TGF-β axis was proposed to represent an important signaling pathway in the primary tumor microenvironment for different types of cancer [41]. GARP is thus a target of interest for immunotherapeutic approaches. A blocking anti-GARP:TGF-β1 mAb, which blocks the release of active TGF-β1 without inducing an antibody-dependent cellular toxicity (ADCC)- or antibody-dependent cellular phagocytosis (ADCP)-mediated depletion of GARP expressing cells, is currently tested in clinical trials in patients with advanced or metastatic solid tumors (NCT03821935 and NCT05822752). Yet, GARP expression in LNs has received very little attention despite the important role of this organ in the elaboration of the immune response. To fill this gap, we examined *LRRC32* and GARP expression by non-immune cells, including endothelial cells constituting different vascular structures (LECs, BECs, and HEVs) and fibroblastic/reticular cells forming a mesh of collagen fibers. Our observations that these non-immune and non-cancerous cells in metastatic LNs can express GARP may suggest that antibodies blocking TGF-β1 activation without inducing the depletion of GARP-expressing cells might bear less risks of undesired side effects that could be associated with a reduction in Treg numbers and destruction of vascular structures.

Our study has some limitations related to technical issues. First, anti-human GARP mAbs are suitable for staining frozen tissue sections but not on formalin-treated paraffin-embedded sections. This restricts analyses to a limited number of human tissue samples. Second, we lack an anti-murine GARP mAb that is suitable for IHC analyses in the pre-clinical model. We overcame this difficulty by conducting in situ mRNA hybridization to detect *Lrrc32* using the RNAscope approach, combined with multiple immunostainings of other markers, but this did not allow for quantitative analyses. Third, the depth of scRNA-Seq makes it possible to visualize only the most highly expressed genes in each cluster, and GARP appeared expressed at a low level in non-immune cells.

Recently, scRNA-Seq analyses have highlighted the heterogeneity of endothelial and fibroblastic cells in LNs [30,31,32,34]. This holds particularly true for human LECs. Indeed, human LECs were separated into eight different subtypes, among which three subtypes are located in the SCS, namely ceiling LECs (cLECs), floor LECs (fLECs), and bridge LECs (bLECs) located between cLECs and fLECS [31]. These scRNA-Seq data revealed very low levels or no *LRRC32* expression by all the different LEC subpopulations. In contrast, GARP expression at the protein level was found around lymphatic vessels (CD34-, PDPN+) in human samples. It is worth noting that protein GARP was detected in both fLECs and cLECs in the SCS. In the murine scRNA-Seq dataset, mRNA *Lrrc32* was detected in the LEC I cluster corresponding to ceiling, collector, and valve LECs. *Lrrc32* mRNA expression in murine LECs was confirmed by RNAscope imagery (Prox1+/Lrrc32+ cells) in both parenchymal LECs and in the SCS. Lrcc32 mRNA was found in Lyve1^low^/Prox1+ cLECs and Lyve1+/Prox1+ fLECs, delineating the SCS. This is in line with the data obtained with human LN tissue sections. The cLECs expressed several matrix proteins deposited close to the collagenous matrix of the LN capsule [40]. It is currently considered that fLECs could serve as a receptive surface for antigen-presenting cells entering the SCS by the afferent lymph [42]. Our data raise the possibility that GARP plays a role in the SCS, a specialized area of the LN involved in the entrance of immune and/or cancer cells, as well as soluble tumor antigens derived from the primary tumor. The apparent discrepancy between the scRNA-Seq data (lack or faint Lrrc32 mRNA detection in LECs) and LN tissue section analysis (IHC and RNAscope revealing GARP and Lrrc32 expression in LECs, respectively) may be related, at least, to the sequencing depth of the datasets.

The analyses of the human and murine scRNA-Seq datasets confirmed the known *LRRC32*/*Lrcc32* expression by BECs [21,25] and revealed expression by different types of BECs forming large arteries and veins, capillaries, and HEVs. Accordingly, our IHC analyses performed on human and murine tissue samples confirmed the expression of protein GARP by BECs (CD34+/PDPN− in human, CD31+/Prox1− in mice). In line with previous studies [21,25], we also confirmed the expression of the *LRRC32* mRNA and the presence of the GARP protein at the surface of human BECs primary cells (HUVECs). Interestingly, we provide for the first time evidence for GARP expression in PNAd+ HEVs in human and mouse samples. These specialized vessels are involved in the entrance of circulating lymphocytes in the LN and the exit of metastatic cells from the LN to distant organs [8,9]. The functional importance of HEV-associated GARP in the control of immune responses and in the metastatic process is still to be elucidated. GARP in HEVs could play a role in the escape of metastatic cells from immune surveillance.

A striking finding from scRNA-Seq data mining is the detection of a particular subpopulation of perivascular cells (PvCs) expressing ITGA7, αSMA, and GARP. Integrin α7β1, comprising ITGA7 and the ITGB1 subunit, is the primary receptor for laminin on skeletal myoblasts and adult myofibers [43]. It is also produced by vascular smooth muscle cells. Notably, α7 null mice that survive to birth exhibit vascular smooth muscle defects [44]. In our IHC analyses of LNs, we found only a few perivascular cells positive for ITGA7. In sharp contrast, an important population of αSMA+ cells surrounding blood vessels was detected and they expressed GARP. These perivascular cells are likely pericytes and/or smooth muscle cells. This perivascular distribution of GARP around blood vessels is intriguing and raises the question of the function of GARP in these areas. One could postulate that GARP exerts a “shielding” role around vessels involved in the entrance and/or exit of immune/metastatic cells.

The sometimes-extended GARP immunoreactivity in the extracellular matrix surrounding vessels (particularly blood vessels) suggests that fibroblastic cells also contribute to GARP production. Accordingly, we found GARP expression by human primary lymphatic fibroblasts (HLFs) in culture. Notably, GARP staining was also detected in parenchymal fibroblastic cells. The presence of GARP in the cLECs adjacent to the LN capsule, together with GARP expression by matrix-associated αSMA+ cells in the parenchyma, points to a putative relationship between GARP and matrix-producing cells. Although the *Lrrc32* mRNAs were detected by RNAscope in those cells, we cannot exclude the possibility that a soluble form of GARP (sGARP) is shed from the surface of these cells or other surrounding cells and deposited in the ECM. The shedding of sGARP from the cell surface of Tregs and platelets has been previously reported [45], and sGARP was reported to exert immunosuppressive properties [21]. The matrix could thus constitute a reservoir for sGARP bound to latent TGF-β1, which could be released during tissue remodeling associated with inflammatory and metastatic processes. However, whether active TGF-β1 can be released from sGARP:TGF-β1 complexes stored in the matrix is speculative and remains to be demonstrated. Indeed, the GARP transmembrane domain and anchorage of GARP:TGF-β1 complexes at the cell surface were shown to be required for TGF-β1 activation by integrins [46]. One cannot exclude TGF-β-independent functions for sGARP in the matrix.

Our data support the concept that GARP could mediate functions of cells other than Tregs [21]. Activated B cells and platelets were reported to express GARP:TGF-β1 complexes and produce active TGF-β1 in a GARP-dependent manner [23,47,48]. In mice, the GARP:TGF-β1 axis in B cells was shown to be a key factor for immune tolerance and the prevention of lupus-like autoimmune diseases [49] and, in platelets, it was shown to play a role in the immune evasion of cancer cells [47]. In multipotent mesenchymal stromal cells (MSCs), GARP was shown to be involved in their resistance to DNA damage and apoptosis in a TGF-β1 dependent manner, as well as in their immunomodulatory activities [50]. Finally, it was recently shown that GARP expressed on hepatic stellate cells drives the development of liver fibrosis via the activation of latent TGF-β1 [24].

The use of cell-specific *Lrrc32* KO mice provided evidence that targeting Garp on Tregs, but not on platelets, with a blocking anti-GARP:TGF-β1 antibody induced tumor regression and overcame resistance to PD1 blockade in tumor-bearing mice [40]. Indeed, the blocking anti-GARP:TGF-β1 mAb exerted anti-tumor efficacy in platelets-specific *Lrrc32* KO mice, but lost its activity in Tregs-specific *Lrrc32* KO mice. The activity of TGF-β1 produced by GARP-expressing Tregs was thus required for anti-GARP:TGF-β1 to exert anti-tumor activity. These findings also suggested that blocking the action of TGF-β1 emanating from GARP-expressing platelets or endothelial cells was neither necessary nor sufficient, at least for primary tumor progression. In vitro, under basal culture conditions, GARP and the αVβ6 and αVβ8 integrins were detected at the surface of HUVECs, LECs, and HLFs by flow cytometry. Despite several attempts in different experimental settings, we failed to observe TGF-β1 activation in BEC, HLF, and LEC cultures. This is in line with the previous study of Bertrand et al. [25]. Thus, functional studies remain essential to determine the GARP function, if any, in endothelial and fibroblastic cells in LNs. Due to the high levels of GARP protein detected in the LNs, one is expecting a function(s) that remain(s) to be determined.

## 5. Conclusions

The identity, spatial distribution, and cellular sources of GARP-expressing cells in normal and metastatic LNs have remained elusive. Here, we provide the first mapping of GARP expression in human and murine metastatic LNs. In addition to confirming GARP expression in BECs and Tregs in LNs, our data provide striking evidence for GARP production by specialized LEC subtypes in the SCS (cLECs and fLECs) in HEVs and matrix-associated (fibroblastic/perivascular) cells. Our findings suggest a role for GARP in two vascular structures localized at the interface between the LN and the afferent/blood vessels, as well as in matrix-associated cells, that is worth considering for future studies.

## Data Availability

All data are available in the manuscript.

## Supplementary Materials

The following supporting information can be downloaded at: https://www.mdpi.com/article/10.3390/cancers15235621/s1. Figure S1. Multiplex immunofluorescence identifies GARP expression by Tregs and non-Tregs in human LN.

## References

[1] Chatterjee G., Pai T., Hardiman T., Avery-Kiejda K., Scott R.J., Spencer J., Pinder S.E., Grigoriadis A. (2018). Molecular Patterns of Cancer Colonisation in Lymph Nodes of Breast Cancer Patients. Breast Cancer Res..

[2] Balsat C., Blacher S., Herfs M., Van de Velde M., Signolle N., Sauthier P., Pottier C., Gofflot S., De Cuypere M., Delvenne P. (2017). A Specific Immune and Lymphatic Profile Characterizes the Pre-Metastatic State of the Sentinel Lymph Node in Patients with Early Cervical Cancer. Oncoimmunology.

[3] Wakisaka N., Hasegawa Y., Yoshimoto S., Miura K., Shiotani A., Yokoyama J., Sugasawa M., Moriyama-Kita M., Endo K., Yoshizaki T. (2015). Primary Tumor-Secreted Lymphangiogenic Factors Induce Pre-Metastatic Lymphvascular Niche Formation at Sentinel Lymph Nodes in Oral Squamous Cell Carcinoma. PLoS ONE.

[4] Tammela T., Alitalo K. (2010). Lymphangiogenesis: Molecular Mechanisms and Future Promise. Cell.

[5] Maus R.L.G., Jakub J.W., Hieken T.J., Nevala W.K., Christensen T.A., Sutor S.L., Flotte T.J., Markovic S.N. (2019). Identification of Novel, Immune-Mediating Extracellular Vesicles in Human Lymphatic Effluent Draining Primary Cutaneous Melanoma. OncoImmunology.

[6] Cho J.K., Hyun S.H., Choi N., Kim M.J., Padera T.P., Choi J.Y., Jeong H.S. (2015). Significance of Lymph Node Metastasis in Cancer Dissemination of Head and Neck Cancer. Transl. Oncol..

[7] Stacker S.A., Williams S.P., Karnezis T., Shayan R., Fox S.B., Achen M.G. (2014). Lymphangiogenesis and Lymphatic Vessel Remodelling in Cancer. Nat. Rev. Cancer.

[8] Brown M., Assen F.P., Leithner A., Abe J., Schachner H., Asfour G., Bago-Horvath Z., Stein J.V., Uhrin P., Sixt M. (2018). Lymph Node Blood Vessels Provide Exit Routes for Metastatic Tumor Cell Dissemination in Mice. Science.

[9] Padera T.P., Meijer E.F.J., Munn L.L. (2016). The Lymphatic System in Disease Processes and Cancer Progression. Annu. Rev. Biomed. Eng..

[10] Sleeman J.P. (2015). The Lymph Node Pre-Metastatic Niche. J. Mol. Med..

[11] Kaplan R.N., Riba R.D., Zacharoulis S., Bramley A.H., Vincent L., Costa C., MacDonald D.D., Jin D.K., Shido K., Kerns S.A. (2005). VEGFR1-Positive Haematopoietic Bone Marrow Progenitors Initiate the Pre-Metastatic Niche. Nature.

[12] Peinado H., Zhang H., Matei I.R., Costa-Silva B., Hoshino A., Rodrigues G., Psaila B., Kaplan R.N., Bromberg J.F., Kang Y. (2017). Pre-Metastatic Niches: Organ-Specific Homes for Metastases. Nat. Rev. Cancer.

[13] Psaila B., Lyden D. (2009). The Metastatic Niche: Adapting the Foreign Soil. Nat. Rev. Cancer.

[14] Hirakawa S., Kodama S., Kunstfeld R., Kajiya K., Brown L.F., Detmar M. (2005). VEGF-A Induces Tumor and Sentinel Lymph Node Lymphangiogenesis and Promotes Lymphatic Metastasis. J. Exp. Med..

[15] Hirakawa S., Brown L.F., Kodama S., Paavonen K., Alitalo K., Detmar M. (2007). VEGF-C–Induced Lymphangiogenesis in Sentinel Lymph Nodes Promotes Tumor Metastasis to Distant Sites. Blood.

[16] Gillot L., Lebeau A., Baudin L., Pottier C., Louis T., Durré T., Longuespée R., Mazzucchelli G., Nizet C., Blacher S. (2022). Periostin in Lymph Node Pre-Metastatic Niches Governs Lymphatic Endothelial Cell Functions and Metastatic Colonization. Cell. Mol. Life Sci..

[17] De Streel G., Lucas S. (2021). Targeting Immunosuppression by TGF-Β1 for Cancer Immunotherapy. Biochem. Pharmacol..

[18] Travis M.A., Sheppard D. (2014). TGF-β Activation and Function in Immunity. Annu. Rev. Immunol..

[19] Liénart S., Merceron R., Vanderaa C., Lambert F., Colau D., Stockis J., Van Der Woning B., De Haard H., Saunders M., Coulie P.G. (2018). Structural Basis of Latent TGF-Β1 Presentation and Activation by GARP on Human Regulatory T Cells. Science.

[20] Cuende J., Liénart S., Dedobbeleer O., Van Der Woning B., De Boeck G., Stockis J., Huygens C., Colau D., Somja J., Delvenne P. (2015). Monoclonal Antibodies against GARP/TGF-Β1 Complexes Inhibit the Immunosuppressive Activity of Human Regulatory T Cells In Vivo. Sci. Transl. Med..

[21] Zimmer N., Trzeciak E.R., Graefen B., Satoh K., Tuettenberg A. (2022). GARP as a Therapeutic Target for the Modulation of Regulatory T Cells in Cancer and Autoimmunity. Front. Immunol..

[22] Tran D.Q., Andersson J., Wang R., Ramsey H., Unutmaz D., Shevach E.M. (2009). GARP (LRRC32) Is Essential for the Surface Expression of Latent TGF-Beta on Platelets and Activated FOXP3+ Regulatory T Cells. Proc. Natl. Acad. Sci. USA.

[23] Dedobbeleer O., Stockis J., Van Der Woning B., Coulie P.G., Lucas S. (2017). Cutting Edge: Active TGF-Β1 Released from GARP/TGF-Β1 Complexes on the Surface of Stimulated Human B Lymphocytes Increases Class-Switch Recombination and Production of IgA. J. Immunol..

[24] Zhang X., Sharma P., Maschmeyer P., Hu Y., Lou M., Kim J., Fujii H., Unutmaz D., Schwabe R.F., Winau F. (2023). GARP on Hepatic Stellate Cells Is Essential for the Development of Liver Fibrosis. J. Hepatol..

[25] Bertrand C., Van Meerbeeck P., De Streel G., Vaherto-Bleeckx N., Benhaddi F., Rouaud L., Noël A., Coulie P.G., Van Baren N., Lucas S. (2021). Combined Blockade of GARP:TGF-Β1 and PD-1 Increases Infiltration of T Cells and Density of Pericyte-Covered GARP+ Blood Vessels in Mouse MC38 Tumors. Front. Immunol..

[26] Vermeersch E., Denorme F., Maes W., De Meyer S.F., Vanhoorelbeke K., Edwards J., Shevach E.M., Unutmaz D., Fujii H., Deckmyn H. (2017). The Role of Platelet and Endothelial GARP in Thrombosis and Hemostasis. PLoS ONE.

[27] Gillot L., Baudin L., Rouaud L., Kridelka F., Noël A. (2021). The Pre-Metastatic Niche in Lymph Nodes: Formation and Characteristics. Cell. Mol. Life Sci..

[28] Miyasaka M., Hata E., Tohya K., Hayasaka H. (2016). Lymphocyte Recirculation. Encyclopedia of Immunobiology.

[29] Acton S.E., Onder L., Novkovic M., Martinez V.G., Ludewig B. (2021). Communication, Construction, and Fluid Control: Lymphoid Organ Fibroblastic Reticular Cell and Conduit Networks. Trends Immunol..

[30] Rodda L.B., Lu E., Bennett M.L., Sokol C.L., Wang X., Luther S.A., Barres B.A., Luster A.D., Ye C.J., Cyster J.G. (2018). Single-Cell RNA Sequencing of Lymph Node Stromal Cells Reveals Niche-Associated Heterogeneity. Immunity.

[31] Abe Y., Sakata-Yanagimoto M., Fujisawa M., Miyoshi H., Suehara Y., Hattori K., Kusakabe M., Sakamoto T., Nishikii H., Nguyen T.B. (2022). A Single-Cell Atlas of Non-Haematopoietic Cells in Human Lymph Nodes and Lymphoma Reveals a Landscape of Stromal Remodelling. Nat. Cell Biol..

[32] Takeda A., Hollmén M., Dermadi D., Pan J., Brulois K.F., Kaukonen R., Lönnberg T., Boström P., Koskivuo I., Irjala H. (2019). Single-Cell Survey of Human Lymphatics Unveils Marked Endothelial Cell Heterogeneity and Mechanisms of Homing for Neutrophils. Immunity.

[33] Xiang M., Grosso R.A., Takeda A., Pan J., Bekkhus T., Brulois K., Dermadi D., Nordling S., Vanlandewijck M., Jalkanen S. (2020). A Single-Cell Transcriptional Roadmap of the Mouse and Human Lymph Node Lymphatic Vasculature. Front. Cardiovasc. Med..

[34] Fujimoto N., He Y., D’Addio M., Tacconi C., Detmar M., Dieterich L.C. (2020). Single-Cell Mapping Reveals New Markers and Functions of Lymphatic Endothelial Cells in Lymph Nodes. PLoS Biol..

[35] Li L., Shirkey M.W., Zhang T., Piao W., Li X., Zhao J., Mei Z., Guo Y., Saxena V., Kensiski A. (2022). Lymph Node Fibroblastic Reticular Cells Preserve a Tolerogenic Niche in Allograft Transplantation through Laminin A4. J. Clin. Investig..

[36] Stockis J., Colau D., Coulie P.G., Lucas S. (2009). Membrane Protein GARP Is a Receptor for Latent TGF-β on the Surface of Activated Human Treg: Cellular Immune Response. Eur. J. Immunol..

[37] Bankhead P., Loughrey M.B., Fernández J.A., Dombrowski Y., McArt D.G., Dunne P.D., McQuaid S., Gray R.T., Murray L.J., Coleman H.G. (2017). QuPath: Open Source Software for Digital Pathology Image Analysis. Sci. Rep..

[38] Chang J.E., Turley S.J. (2015). Stromal Infrastructure of the Lymph Node and Coordination of Immunity. Trends Immunol..

[39] Van De Velde M., García-Caballero M., Durré T., Kridelka F., Noël A., Cal S., Obaya A.J. (2018). Ear Sponge Assay: A Method to Investigate Angiogenesis and Lymphangiogenesis in Mice. Proteases and Cancer.

[40] De Streel G., Bertrand C., Chalon N., Liénart S., Bricard O., Lecomte S., Devreux J., Gaignage M., De Boeck G., Mariën L. (2020). Selective Inhibition of TGF-Β1 Produced by GARP-Expressing Tregs Overcomes Resistance to PD-1/PD-L1 Blockade in Cancer. Nat. Commun..

[41] Lahimchi M.R., Eslami M., Yousefi B. (2022). New Insight into GARP Striking Role in Cancer Progression: Application for Cancer Therapy. Med. Oncol..

[42] Jalkanen S., Salmi M. (2020). Lymphatic Endothelial Cells of the Lymph Node. Nat. Rev. Immunol..

[43] Gerli M.F.M., Moyle L.A., Benedetti S., Ferrari G., Ucuncu E., Ragazzi M., Constantinou C., Louca I., Sakai H., Ala P. (2019). Combined Notch and PDGF Signaling Enhances Migration and Expression of Stem Cell Markers While Inducing Perivascular Cell Features in Muscle Satellite Cells. Stem Cell Rep..

[44] Flintoff-Dye N.L., Welser J., Rooney J., Scowen P., Tamowski S., Hatton W., Burkin D.J. (2005). Role for the A7β1 Integrin in Vascular Development and Integrity. Dev. Dyn..

[45] Hahn S.A., Stahl H.F., Becker C., Correll A., Schneider F.-J., Tuettenberg A., Jonuleit H. (2013). Soluble GARP Has Potent Antiinflammatory and Immunomodulatory Impact on Human CD4+ T Cells. Blood.

[46] Wang R., Zhu J., Dong X., Shi M., Lu C., Springer T.A. (2012). GARP Regulates the Bioavailability and Activation of TGFβ. Mol. Biol. Cell.

[47] Metelli A., Wu B.X., Riesenberg B., Guglietta S., Huck J.D., Mills C., Li A., Rachidi S., Krieg C., Rubinstein M.P. (2020). Thrombin Contributes to Cancer Immune Evasion via Proteolysis of Platelet-Bound GARP to Activate LTGF-β. Sci. Transl. Med..

[48] Lecomte S., Devreux J., de Streel G., van Baren N., Havelange V., Schröder D., Vaherto N., Vanhaver C., Vanderaa C., Dupuis N. (2023). Therapeutic Activity of GARP:TGF-Β1 Blockade in Murine Primary Myelofibrosis. Blood.

[49] Wallace C.H., Wu B.X., Salem M., Ansa-Addo E.A., Metelli A., Sun S., Gilkeson G., Shlomchik M.J., Liu B., Li Z. (2018). B Lymphocytes Confer Immune Tolerance via Cell Surface GARP-TGF-β Complex. JCI Insight.

[50] Carrillo-Gálvez A.B., Quintero J.E., Rodríguez R., Menéndez S.T., Victoria González M., Blanco-Lorenzo V., Allonca E., De Araújo Farias V., González-Correa J.E., Erill-Sagalés N. (2020). GARP Promotes the Proliferation and Therapeutic Resistance of Bone Sarcoma Cancer Cells through the Activation of TGF-β. Cell Death Dis..

