# Supplementary material for: Spatial Distribution of Non-Immune Cells Expressing Glycoprotein A Repetitions Predominant in Human and Murine Metastatic Lymph Nodes
Source: Cancers (Basel). 2023 Nov 28;15(23):5621. doi: 10.3390/cancers15235621 (PMC10705676; doi:10.3390/cancers15235621)
Supplement: Supplementary file 1 [file cancers-15-05621-s001.zip › cancers-2718622-supplementary.pdf]

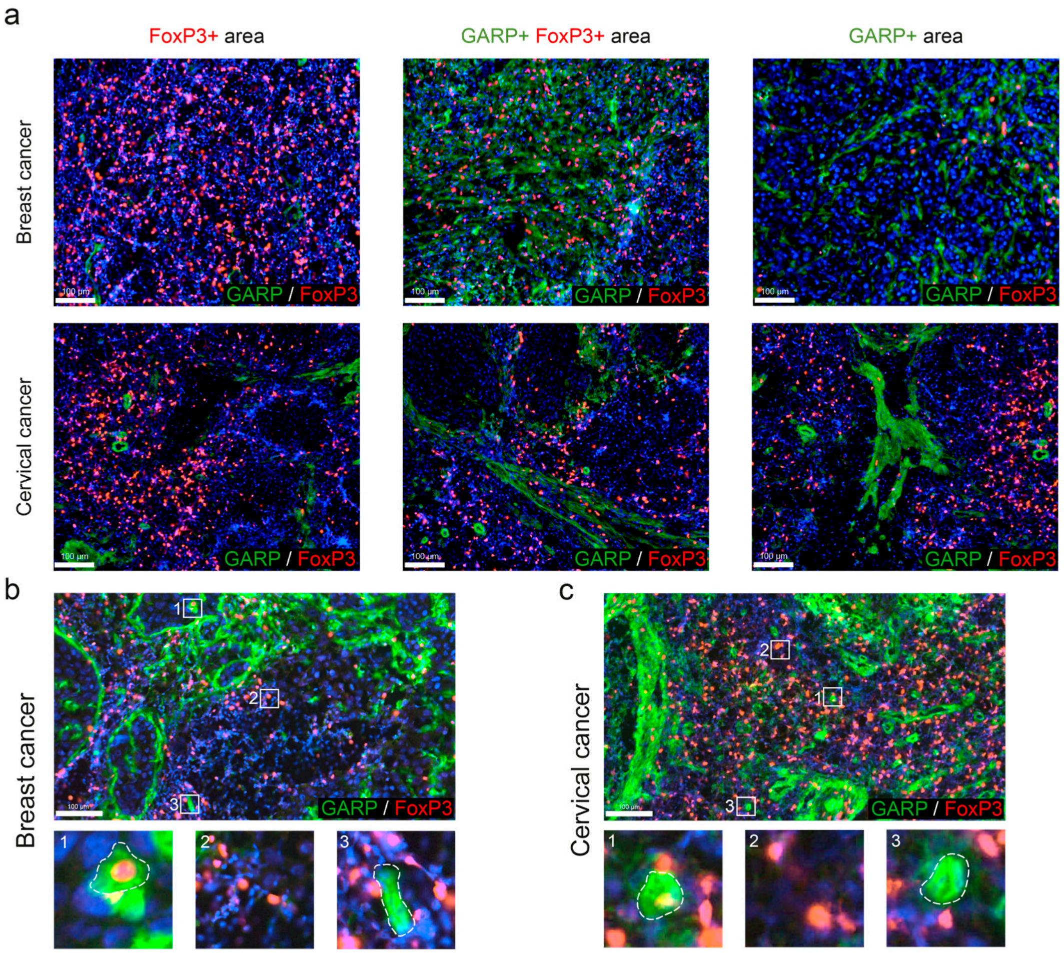

Figure S1. Multiplex immunofluorescence identifies GARP expression by Tregs and non-Tregs in human LN. (**a**) Dual-plex immunofluorescence staining of GARP (in green), FOXP3 (in red), and nuclei (DAPI, in blue) on MLN+ from patients with breast cancer or cervical cancer. (**b, c**) Focus on FoxP3/GARP pattern, scale bar = 100 µm and higher magnification of (1) GARP+/FOXP3 cells, (2) GARP-/FOXP3+ cells, and (3) GARP+/FOXP3- cells in (**b**) breast cancer or (**c**) cervical cancer.
